# Nanomaterials in consumer products: a challenging analytical problem

**DOI:** 10.3389/fchem.2015.00048

**Published:** 2015-08-06

**Authors:** Catia Contado

**Affiliations:** Department of Chemical and Pharmaceutical Sciences, University of FerraraFerrara, Italy

**Keywords:** nanoparticles, silicon dioxide, titanium dioxide, zinc oxide, silver nanoparticles, food products, cosmetic products

## Abstract

Many products used in everyday life are made with the assistance of nanotechnologies. Cosmetic, pharmaceuticals, sunscreen, powdered food are only few examples of end products containing nano-sized particles (NPs), generally added to improve the product quality. To evaluate correctly benefits vs. risks of engineered nanomaterials and consequently to legislate in favor of consumer's protection, it is necessary to know the hazards connected with the exposure levels. This information implies transversal studies and a number of different competences. On analytical point of view the identification, quantification and characterization of NPs in food matrices and in cosmetic or personal care products pose significant challenges, because NPs are usually present at low concentration levels and the matrices, in which they are dispersed, are complexes and often incompatible with analytical instruments that would be required for their detection and characterization. This paper focused on some analytical techniques suitable for the detection, characterization and quantification of NPs in food and cosmetics products, reports their recent application in characterizing specific metal and metal-oxide NPs in these two important industrial and market sectors. The need of a characterization of the NPs as much as possible complete, matching complementary information about different metrics, possible achieved through validate procedures, is what clearly emerges from this research. More work should be done to produce standardized materials and to set-up methodologies to determine number-based size distributions and to get quantitative date about the NPs in such a complex matrices.

## Introduction

The first known use of the word “nanotechnology” dates back to 1974 when prof. Norio Taniguchi coined the term to describe semiconductor processes (Taniguchi, 1974). Since then the term has assumed a futuristic undertone and the nanoparticles (NPs), one of the “building blocks” of nanotechnology, have been seen as stranger entities even if natural NPs have been around us since ever. Starting form 1980s, the term nanotechnology refers to the fabrication, use/manipulation, control and characterization of structures devices or materials with a least one dimension in the size range of 1–100 nm (1 nm–10^−9^ m) (SCENHIR, 2007a; ISO/TS 27687, 2008).

Nanotechnologies represent a fast-growing market, which brings a combination of benefits, promises, risks, and uncertainties. Nanotechnologies have improved, and in some cases revolutionized, many industrial sectors, some of which are very close to our everyday life such as the agriculture, food safety, medicine, pharmacy, cosmetic, and personal care.

Focusing on NPs, they might be classified as natural and anthropogenic, these latter, additionally distinguished in two general categories: incidental and engineered (ENPs). Incidental NPs are the byproducts of human activities, generally have poorly controlled sizes and shapes, and may be made of a hodge-podge of different elements, ENPs (polymeric-organic and inorganic) are on the contrary, specifically designed and deliberately synthesized by human beings.

The recent concern about the hazard of NPs is linked essentially to their dimensions, compatible and comparable with most living cell systems. In principle, a product containing nanomaterials or involving the application of nanotechnology cannot be judged as intrinsically harmful (or benign) (FDA, 2014). The dangerousness of a certain new nanomaterial, or certain ENPs, should be evaluated considering not only its application, but also including the production, the use and the final disposal. Some ENPs could be virtually non-toxic, in reason of their composition, size and morphology, or if they are contained in coatings, microelectronics, and other engineered materials, but each product leads to different potential exposures, posing therefore different potential hazards (SCENHIR, 2007b; Mitrano et al., 2015).

By considering the plethora of the possible application sectors, the list of the ENPs that could be dispersed in the environment and create concern is very long, but it is obvious that not all of them have the same impact on the market. The categories of nanomaterials, which move the largest market volume include inorganic non-metallic nanomaterials, carbon based nanomaterials metal NPs and organic, macromolecular or polymeric particulate materials (Table 1). In terms of industrial impact and public exposure, the above-mentioned nanomaterials are in the list of prioritized materials that require an immediate regulatory attention (OCDE, 2009a; Commission staff working paper (EU), 2012; EC Memo, 2012).

**Table 1:** **List of representative manufactured nanomaterial for testing (OCDE, 2009a; EC Memo, 2012)**.

**Type of NPs**		**Food additive**	**EC Number**	**CAS Number**
Inorganic non-metallic nanomaterials	synthetic amorphous silica, SiO_2_, aluminum oxide, Al_2_O_3_ titanium dioxide, TiO_2_, (Anatase) titanium dioxide, TiO_2_, (Rutile) titanium dioxide, TiO_2_ zinc oxide, ZnO, cerium oxide, CeO_2_ iron nanoparticles, Fe_2_O_3_ iron nanoparticles Fe_3_O_4_ nanoclays	E 551 E 171 E 175	231-545-4 215-691-6 215-280-1 215-282-2 215-222-5 236-675-5 215-150-4 15-168-2 215-277-5	1344-28-1 1317-70-0 1317-80-2 1314-13-2 13463-67-7
Carbon based nanomaterials	carbon black, carbon nanotubes, fullerenes C60, multi-walled carbon nanotubes MWCNTs, single-walled carbon nanotubes SWCNTs		215-609-9	1333-86-4
Metal NPs	nanosilver	E 174	231-131-3	231-131-3
Organic, macromolecular or polymeric particulate materials	dendrimers, polystyrene			

All Countries have Regulatory Agencies guarantors of the public health. They are appointed to speed product innovations and to regulate the use of products. In the case of nanotechnological products, their assignment t is not simple, since they have to make regulatory decisions about products ranging from chemotherapy agents to cosmetics, passing through drugs or food products, each in accordance with the specific legal standards applicable to each type of product under its jurisdiction (FDA website^1^).

The USA Regulatory Agencies have focused, for now, their attention only to the few types of nanotechnology-enabled goods in use daily by consumers, such as electronics, batteries, clothing, sporting equipment, food, food packaging, dietary supplements, cosmetics, personal care products, drugs, and medical products.

Analogously is doing the European Commission, which is taking care, among many others, of food and food supplements, pharmaceutical, medicinal products, cosmetics, and personal care products, focusing the attention mainly to some types of ENPs (Table 1) (Commission staff working paper (EU), 2012), but foreseeing to extend the current list to other materials, that after evaluation presented particles of dimensions around the lower size limit of the EU definition of nanomaterial (Commission Recommendation, 2011).

Respect to the definition of “*nanomaterial*,” in a time of economical globalization, where the production and distribution of goods occurs on a world scale market, a harmonized definition among all Countries would be desirable. Unfortunately, many standardization Organization have produced their own definition, depending on their scope and the type of applications they intend to address (scientific, regulatory, industrial) (Bleeker et al., 2013; Rauscher et al., 2014; Roebben et al., 2014). For example, FDA, which has not established regulatory definitions of “nanotechnology,” “nanomaterial,” and “nanoscale,” in June 2014 issued a guidance for industry titled “Considering Whether an FDA-Regulated Product Involves the Application of Nanotechnology” (FDA, 2013) as a reference document. The European Commission, in 2011; recommended the following definition: “*Nanomaterial means a natural, incidental or manufactured material containing particles, in an unbound state or as an aggregate or as an agglomerate and where, for 50% or more of the particles in the number size distribution, one or more external dimensions is in the size range 1–100 nm*” (Commission Recommendation, 2011). This Commission Recommendation has been revised and updated in 2014 (Rauscher et al., 2014; Roebben et al., 2014). The Commission Recommendation has not a legal value, but within the European Union, it aimed at harmonizing the existing and future legislation in a regulatory and policy context. The definition, explicitly limited to particulate materials, includes *de facto* all materials regardless from their origin or purpose (Roebben et al., 2014).

It is important to highlight that, after the publication of the EC definition, some member Countries have introduced a record and classification of the NPs produced and employed within the EC territory. France was the first that has introduced such a register (January 1st 2013, website www.r-nano.fr), where manufacturers have to identify the use of “substances with nanoparticle status” that they produce, import, distribute, or formulate (required by Articles L. 523-1 to L. 523-5 of the French Environmental Code). Denmark, Belgium and Germany instead have included the nanomaterial definition in their legislative provisions, by restricting the scope, for example by origin, or to certain groups of substances and products (Powers et al., 2006).

This paper, based mainly on the literature published in last 5 years, aims to recalls some of the analytical techniques which might be useful in the physico-chemical characterization of NPs employed in the food and in the cosmetic field, and reports their most recent applications.

### Which characteristics of a nanoparticle matter?

Nanomaterials have usually physical, chemical, or biological properties that are different from those of larger scale material with the same chemical composition. Such differences may include altered magnetic properties, altered electrical or optical activity, increased structural integrity, or altered chemical or biological activity (FDA, 2013).

To determine if a substance produced with nanotechnology is safe for the proposed use, or if it has possible health or/and environmental effects (transport, fate, interaction with living organisms) a number of physical and chemical parameters should be evaluated. These include size and shape, state of dispersion, physical and chemical properties, surface area, and surface chemistry (Powers et al., 2006). Size, shape and more in general the morphology are surely the principal characteristics, which affect the toxicity of NPs by affecting where they depose, the clearance from the body, and the biological responses, such as inflammation. However, the way in which NPs interact with the organisms or the environment depends mainly on the surface area and the surface chemistry, which both determine also the particle dispersion characteristics and the adsorption of ions and biomolecules. This means that either the physical form and the chemical reactivity should be equally evaluated, since both are equally important (Oberdorster et al., 2005; Powers et al., 2006; OECD, 2009b, 2010; FDA, 2014).

An exhaustive characterization of NPs is often time consuming, expensive and complex, in addition it could happen that laboratories are not equipped with all the necessary facilities and do not have the correspondent appropriate competences. The objectives of the study (for example, the optimization for production and quality assurance or the interaction with biological systems) determine in most cases the type of characterization required, nevertheless, there are a number of fundamental and essential properties that should be measured, regardless the study purposes (Table 2). The order of priority for the required metrics and the collaboration with laboratories that possess the opportune theoretical and technical expertise are the keys for a useful NPs characterization.

**Table 2:** **List of the physico-chemical parameters that should be determined for food and cosmetic applications (EFSA, 2011; FDA, 2013)**.

**Physico-chemical parameters**	**Possible techniques**
Chemical composition/Identity	A wide range of analytical methods, including UV -Vis, HPLC, GC/LC -MS, AAS, ICP-MS, FTIR, NMR, XRD, etc
Particle size (primary/secondary) and particle size distribution	FFF, HDC, HPLC, AUC, CPS disc centrifugation, TEM, SEM, AFM, DLS, DMA
Structure: aggregation and agglomeration characteristics	
Particle and mass concentration	
Morphology: shape, surface area, surface topology (roughness), crystallinity, porosity	AFM, TEM, SEM, NMR, XRD, BET
Surface chemistry: zeta potential/surface charge, surface coating, functionalization, catalytic activity	LDE, SPM, XPS, MS, RS, FTIR, NMR, AUC (for surface composition), GE, SPM, LDE, PALS (for zeta potential), Nano SIMS, SERS
Redox potential	Potentiometric methods, X-ray absorption spectroscopy
Solubility	
Dustiness	
Density and Pour Density^*^	
Viscosity	
Stability	MS, HPLC, DLS, FTIR, NMR
Photocatalitic activity	
UV absorption (extinction coefficient), light reflection	UV-Vis

* *The “pour density” is the apparent density of a bed of material formed in a container of standard dimensions when a specified amount of the material is introduced without settling. The “tap density” is the density after the material is vibrated or tapped under standard conditions*.

On consumer prospective, it would be desirable to read on the product's label if a certain product contains an ingredient in a nano-form, especially in the case of food, pharmaceutical, or cosmetic/personal care products. This apparent simple request, charged to the companies, hides in reality, many technical problems. To disclose on the label the presence of an ingredient in the nano-form, this has to be determined and quantified, which means to solve: (1) how to determine the presence of NPs inside of an end product; (2) how to measure the external particle size (1–100 nm); (3) how to determine the number size distribution in order to apply the EC regulatory provisions.

**Figure 1:**
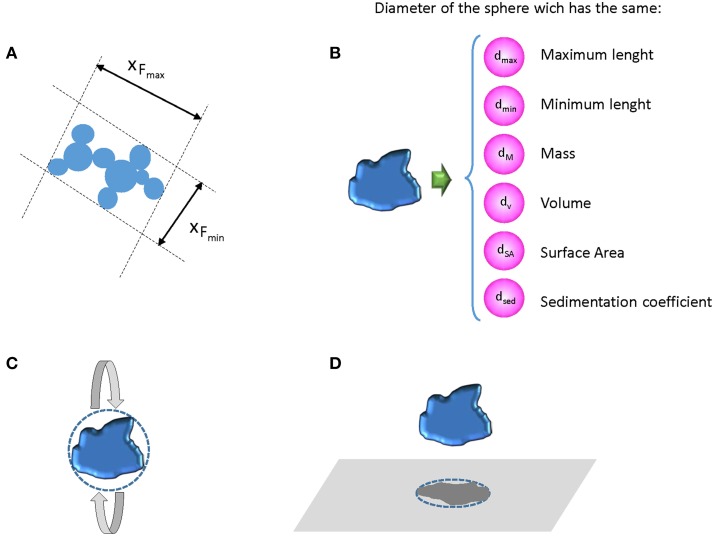
**(A)** Possible external dimensions of an irregular aggregate; **(B)** different diameter expressions when an irregular particle is approximate to a sphere, **(C)** diameter of a sphere that has the same inertia of rotation, and **(D)** diameter of the circle causing the same electro-shadow area (Linsinger et al., 2013).

The term “particle size” it is a challenge itself, since unless particles are perfect spheres, which is rarely the case, every non-spherical particle can be characterized by multiple “sizes,” and these sizes may differ between the dried or dispersed state of a particle (Linsinger et al., 2012) (Figure 1). All methods for particle size analysis target one of these sizes, i.e., each method measures fundamentally different parameters, rendering the measured sizes method-defined properties (Roebben et al., 2010). The term “size of a particle” is so meaningless without specification of the type of size (e.g., hydrodynamic diameter) and the method used to obtain this size information (Linsinger et al., 2013). As a consequence, average sizes and size distributions obtained with different methods might disagree because of the different physical principles behind the measurement and the sample preparation procedure required for the measurement.

Within Europe, the provisions derived from the EC nanomaterial definition foreseen the determination of the number size distribution, justified by the fact that when the NPs are produced, often they do not have all an equal dimension, but often follow rather broad distributions (Ganzleben et al., 2011). Unfortunately, mean particle sizes can be determined with some reliability, particle size distributions cannot (Linsinger et al., 2013).

A set of standard measurement methods suitable for particle size analysis with a number of specific instrumental techniques can be found in “ISO/TC 24/SC 4 Particle characterization” (ISO, website^2^), however, these methods are not written with the specific purpose of meeting the EU nanomaterial definition. Often, the mass or intensity weighted distributions must be converted into number based distributions, through conversion factors known only approximately, which makes such a distributions not fully reliable. Therefore, currently there are no reliable methods available for the determination of accurate number-based size distributions and it will take time before reliable measurement results are obtained in this new measurement area (Roebben et al., 2014).

The problem of achieving a reliable measurement can be overcome by using “validate” methods, but laboratories must have the expertise to implement the methods and to use materials for quality assurance to calibrate and verify the performance of both methods and laboratories. A list of methods suggested for determining the size of NPs is reported in EFSA (2011) and Linsinger et al. (2012). The major issues, these methods have to face, are the presence of aggregates/agglomerates, the working range, and the possibility to define a protocol to validate the method. In the following paragraph, some of them are briefly recalled with the awareness that there is no single sizing technique that is superior in all applications, but that the material, which has to be measured, and its environment determine the best instrument/method choice.

### Techniques to measure the NP sizes and the distributions

A number of publications have listed and described some techniques suitable to characterize the NPs (Luykx et al., 2008; Dhawan and Sharma, 2010; Lopez-Serrano et al., 2014; Valcárcel and López-Lorente, 2014). The Electron Microscopy techniques are still the most popular among the sizing methods, followed by Dynamic Light Scattering (DLS) and X-ray Diffraction (XRD) methods (Table 3); however, there are many others equivalently performing and useful, to choose depending on the sample.

**Table 3:** Literature search on SCOPUS on 25th April 2015—Range time 2010–2015—Searched words Nanoparticles + Sizing methods = 2202 documents^*^.

**Technique**	**No. of papers**	**%**
TEM	234	10.6
SEM	188	8.54
AFM	162	7.36
DLS	152	6.9
XRD	77	3.5
SAXS	56	2.5
HDC	28	1.3
FFF	22	1
BET	13	0.6
CPS	9	0.4
PTA	3	0.1

* *Article (1564), Review (342), Conference Paper (167), Book Chapter (76), Book (30), Conference Review (11), Short Survey (4), Article in Press (2), Letter (2), Note*.

Methods can be grouped mainly in three categories: counting methods, ensemble methods (i.e., methods that measure large number of particles simultaneously), and separation methods (Table 4) (Linsinger et al., 2012; Singh et al., 2014; Szakal et al., 2014).

**Table 4:** **Some sizing techniques (Powers et al., 2006; Lespes and Gigault, 2011)**.

	**Sizing technique**	**Nominal size range**
Counting methods	Electron microscopy (EM)	0.3 nm–several microns
	Atomic force microscopy (AFM)	5 nm–8 μm
	Particle tracking analysis (PTA)	10 nm–2 μm
Ensamble methods	Small-angle X-ray scattering (SAXS)	1 nm–100 nm
	X-ray diffraction (XRD)	
	Dynamic light scattering (DLS)	1 nm–2 μm
Separation methods	Centrifugal particle sedimentation (CPS)	5 nm–40 μm
	Field flow fractionation (FFF)	2 nm–200 μm
	Size exclusion chromatography (SEC)	1 nm–2 μm
	Hydrodynamic Chromatography (HDC)	30 nm–60 μm
	Capillary Electrophoresis	0.1 nm–2 μm
	Specific surface area (BET, titration, diffusion charging)	5 nm–several microns
	Time of flight Mass spectroscopy	1 nm–3 μm (100 to >100 MDa)
	Acoustic Techniques	20 nm–10 μm
	Laser diffraction/Static light scattering	40 nm–3 mm
	Low pressure impacter and electrical low Pressure Impactor (ELP	
	Scanning/differential mobility analysis	

#### Counting methods

Scanning electron microscopy (SEM) and transmission electron microscopy (TEM) are the most widely used technique to measure the size of particles and visualize their morphology. Samples are analyzed under conditions of high vacuum. To obtain particle size distributions with a statistical assurance it is necessary to count a large number of particles. Both techniques are accurate but time-consuming, especially for the sample preparation. Samples containing water or other solvent can be observed by environmental SEM (ESEM) or environmental TEM (ETEM) systems, which allow to maintain a higher vapor pressure around the sample (Lorenz et al., 2010; Dudkiewicz et al., 2011, 2015; Singh et al., 2014). Other alternative EM methods to avoid dehydration artifact are the cryo-SEM and cryo-TEM, which use a cryo-stage in the microscope under high vacuum to keep the NPs frozen (Dudkiewicz et al., 2011; Lapresta-Fernández et al., 2014). Less common, but useful to evaluate thickness and thin layers is the energy filter TEM analysis (Lari and Dudkiewicz, 2014).

The atomic force microscope (AFM) is considered a cost-effective instrument that has several advantages in the characterization of NPs. Developed to overcome the basic drawback of scanning microscopy that images only conducting or semiconducting surfaces, AFM allows to imaging almost any type of surface, including polymers, ceramics, composites, glass, and biological samples. NPs can be characterized in ambient air and in liquid dispersions, and native tissue can be directly observed without prior dehydration; it requires much less laboratory space than TEM/SEM and it is simpler to operate (Dhawan and Sharma, 2010). An AFM offers visualization in three dimensions with vertical resolutions of less than 0.1 nm and X–Y resolutions of around 1 nm. With this resolution, it is possible to view directly single atoms or molecules that have dimensions of a few nanometers. Particles from 1 nm to 8 μm can be measured in a single scan (Scalf and West, 2006). The accuracy of the lateral (x-y) information depends on the shape of the tip (Dhawan and Sharma, 2010). Surface irregularities observed by SEM are absent on AFM inspection (Luykx et al., 2008).

Nanoparticle tracking analysis (NTA) is an ultramicroscopy technique, which utilizes the properties of both light scattering and Brownian motion to obtain the particle size distribution of samples in liquid suspension (Hole et al., 2013; De Temmerman et al., 2014). The advantages of NTA over TEM are the possibility of measuring large amounts of particles (or tracks) increasing the statistical confidence and the avoidance of particle changes due to preparation of the sample. This facilitates the analysis of particles in broadly-distributed samples. In addition, NTA has the potential to use the intensity of light scattered by individual particles to distinguish particles composed of different materials within a given size class (Gallego-Urrea et al., 2011). NTA is a high sensitivity method, which can detect NPs at concentrations as low as low as 10^6^ particle/cm^3^ and allows to evaluate of number size distributions in a size range 10 nm–2 μm.

#### Ensemble methods

DLS, also referred photo-correlation spectroscopy (PCS) and quasi-elastic light scattering (QELS), measures time-dependent fluctuations in scattering intensity produced by particles in Brownian motion, and yields the size of the particle (hydrodynamic diameter) by applying the Stokes–Einstein relation (Luykx et al., 2008; Dhawan and Sharma, 2010). This method is a good choice if size distributions are narrow. The derived sizes are influenced by the presence of dust or agglomerated particles, consequently, particular attention should be given to the dispersion stability during the sizing to avoid to size aggregates rather than individual particles. The sizes obtained by DLS are usually greater than that measured by other techniques (Cascio et al., 2014), probably because the raw measurement is the amount of light scattered by the particles, which has a strong dependence on the size, skewing the average diameter toward larger particle sizes. The DLS gives the particle size in terms of both intensity and number (ISO, 2008; Dhawan et al., 2009).

XRD is non-destructive analytical technique suitable to get information about the crystal structure, chemical composition, and physical properties of materials and thin films. XRD has a good potential for the analysis of nanostructures, because the width and shape of reflection yield information about the substructure of the materials (sizes of crystallites, microstrain of a lattice, dislocation structures, etc.) (Dorofeev et al., 2012; Wang and Geil, 2012). The measurements are usually made on powders containing a very large number of randomly oriented NPs. When the X-ray beam passes through a thin layer of material, its intensity usually decreases because on the absorption and diffraction. By measuring the position and the intensity of the peaks in the powder diffraction pattern with reference spectra, it is possible identify and quantify the material and its crystalline phases. The XRD peak broadening can be correlated with the (average) finite size of the individual crystallites through the Scherrer's equation; this finite size can correspond to a crystal within a solid, or to individual (monocrystalline) particles in a aggregated/agglomerated nanomaterial (Linsinger et al., 2012).

Small-angle X-ray scattering (SAXS) measures the size of particles through the elastic scattering of monochromatic X-rays caused by inhomogeneities in the electron density within a material (or at the particle surface), measured at very low angles (ca. 0.05–5°). Since the scattering angle depends on the wavelength of incident ray and on the particle size on which it is scattered, if the incident monochromatic X-ray has a wavelength between 0.1 and 1 nm, at low angles (< 10°) are measured the X-rays scattered by particles of sizes of 1–100 nm. Advantages and disadvantages are briefly well synthetized by Kirschbrown (2007). The scattering profiles can be used to determine also information about the shapes, the distance and nature of the interactions between the electron density inhomogeneities (Zou and Zhao, 2015). The samples can be liquid or solid. Results are expressed as radii of spheres, cylinders or discs of equivalent scattering properties (Linsinger et al., 2012).

#### Separation methods

There are various techniques suitable to separate NPs; they are particularly indicated when the particles differ for sizes, for densities or are aggregates and/or agglomerates. Each separation technique takes advantage of different particle properties. Many methods separate the particle in a liquid medium and generally, the analyses are quite fast. Among the various separation methods, one might cite centrifugation, centrifugal particle sedimentation (CPS), high-performance liquid chromatography (HPLC), hydrodynamic chromatography (HDC), size-exclusion chromatography (SEC), field-flow fractionation (FFF), capillary electrophoresis (CE), diafiltration and gel electrophoresis (Kowalczyk et al., 2011; Itoh et al., 2014). Some of these methods, are not suitable for measuring NPs according to the EC definition because of their rather poor separation power, such as for example SEC and HDC, but they are anyhow useful as preparative methods, for isolating the NPs (Gray et al., 2012; Linsinger et al., 2012). Some others have the advantageous features of low operating costs and high reliability.

CPS separates particles by size using centrifugal sedimentation in a liquid medium. The particles sediment within an optically clear and rotating disc, where a slight density gradient stabilizes the sedimentation (Nolte et al., 2012). When NPs approach the outside edge of the rotating disc, they scatter a portion of a laser beam that passes through the disc, and the change in light intensity is continuously recorded. The relation between measured sedimentation time and particle size for spherical particles of uniform and known density is simple. The result of the calculation is an equivalent diameter, more specifically the particle's Stokes' diameter (Oppenheimer, 1983; Nadler et al., 2008). The determined particle size is a method-defined parameter since it depends on the particle density (ISO, 2001; Braun et al., 2012). The measurable size ranges go from 5 nm to 40 μm.

Analytical ultracentrifugation (AUC), similarly to CPS, uses the centrifugal acceleration to separate the components of a sample. The two transport processes that take place in the AUC are the sedimentation, governed by particle density and friction, and the diffusion, independent on particle density but dependent on the particle size (Cölfen, 2004; Planken and Cölfen, 2010). Distributions of the sedimentation coefficient, particle size and shape, molar mass and density can be obtained with Ångström resolution for particle sizes spanning the entire colloidal range thanks to the high rotor speeds (up to 60 krpm). For spherical, compact particles, direct calculation of the diameter is possible; in other cases, further assumptions or information may be required. The AUC is considered a high resolution analysis technique; it is an absolute method since no calibration is required; it requires little amounts of substance and solvent, which might be even aggressive or highly viscous chemicals (Schilling, 2015). The AUC can be coupled with a variety of detectors such as single wavelength absorption, interference and fluorescence. The capabilities of AUC are further enhanced by the development of a multiwavelength UV-vis detector (MWL-AUC), which is capable of determining full UV-vis spectra for each detected species (Walter et al., 2014).

The term FFF identifies a family of separation techniques able to separate sample components thanks to the action of a field force applied perpendicularly to a flow, which flows inside of an empty, thin, and long channel. Depending on the different force fields (liquid flows, centrifugal forces, temperature gradients, or gravity fields), one has a different FFF method (Schimpf et al., 2000). FFF is classified sometimes as one-phase chromatography technique, however the advantage of FFF over conventional column chromatography is the ability to separate both soluble and colloidal components over a wide size range as well as sensitive and sticky samples thanks to the absence of a stationary phase. The most popular FFF method for the separation of NPs is the FlowFFF (AF4) (Gigault et al., 2014), often coupled on-line with specific element detectors, such as ICP-MS (Dubascoux et al., 2010; von der Kammer et al., 2011) even if equally suitable could be the centrifugal FFF, also called sedimentation FFF (Contado et al., 2013), which is actually more selective in term of sizes. One of the main limitations of FFF is that the separation process does not distinguish between NPs and aggregates of the same size/mass nor it can distinguish particles with different shapes, which may have the same mean hydrodynamic diameter (Calzolai et al., 2012).

Size-exclusion chromatography (SEC) is able to separate the sample components by their sizes (Podzimek, 2011). The suspension containing the NPs flows inside of a column packed with porous particles. Separation is achieved by the differential exclusion from the pores of the packing material. The principle feature of SEC is its gentle non-adsorptive interaction with the sample. In the case of NPs some inherent problems such as degradation or losses by irreversible adsorption can be very important. Addition of surfactants in the mobile phase may reduce the mentioned adsorption problems but this new agent can result in a lack of separation resolution (Wei et al., 1999).

HDC separates and sizes solutes or particulates in the micron range (30 nm–60 μm) at a high dilution, without being affected by their density (Revillon, 2009; Szakal et al., 2014). The separation takes place in the inter-particle channels of narrow open packed capillaries, or in wider capillaries with non-porous packing materials, that create capillary routes. In HDC, the larger NPs cannot get as close to the separation particles and therefore spend more time in the high-flow region of the flow than smaller particles, resulting in separation between particles according to size; components are so eluted in the order of decreasing size, as in SEC (Langhorst, 1982). The time from sample introduction to arrival at the detector can be calibrated for apparent (equivalent spherical) particle size. The main advantages of HDC are that it is a rapid and convenient method, which allows to obtain a fingerprint of the size distribution with an easy-to-operate instrument, similar to those used in liquid chromatography, at room temperature (Dekkers et al., 2011).

CE measures the electrophoretic mobility of NPs based on their charge and size distribution in the sample, when an external electric field is applied. Ions move toward the electrode of opposite charge. The separation would be achieved by the mobility of the species depending not only on the solvent medium, but also on the charges, sizes, and shapes of the NPs (Geiger et al., 2012). CE requires minimal amounts of samples and chemicals, it is a fast analysis and it generates minimum waste. Its flexibility and versatility are unrivaled and the same instrumentation can be used to separate a diverse range of analytes, both large and small molecules, whether charged or uncharged (Powers et al., 2006; Zhang et al., 2013). Among the disadvantages one might count the poor sensitivity of photometric detectors, which are the most popular among CE detectors, and the amount of the sample used, which requires in the case of NPs rather high concentration (Lopez-Serrano et al., 2014).

### Comments about the particle size distributions and the particle concentration

As already mentioned, each sizing technique report the particle size results in number, volume, weight, surface area, or intensity, depending on the physical principle on which is based the measurement. The conversion between different particle size distributions is possible and often automatically done by the instrument software. However, it is worthwhile to underline that only image analysis is based on number distribution and its conversion to a volume basis is the only accepted because of the generally very low error (Baalousha et al., 2014). On the contrary, the conversion, for example from an intensity to a volume or number distribution is possible, but it should be useful only to compare to other techniques (Baalousha and Lead, 2012).

Another important aspect, which deserves attention is the metric used to express the particle concentration. Mass per unit volume, number for volume unit, surface for volume unit are common units in the field of NPs and microparticle toxicity assessment (Abbott and Maynard, 2010), in particular for *in vitro* systems, the mass, the number, and the surface for volume unit are used because of the difficulty of measuring directly cellular dose (Hinderliter et al., 2010). Most studies compare the effects of the uptake of different sized NPs referring the exposure to the same concentration of particles measured in mass per unit volume, however, it would be important to distinguish whether the reported effects are due to the size or simply to a difference in NP number (Varela et al., 2012), i.e., if exists a critical particle number concentration, below which cellular uptake depends linearly to the particle number concentration (Dong et al., 2015).

In addition, knowing that NP agglomerates can settle and diffuse differentially according to their “hydrodynamic” diameter and effective density, for *in vitro* experiments it would be important an accurate dosimetry (Cohen et al., 2014).

### Techniques to measure the surface area and its composition

The Brunauer, Emmett and Teller (BET) method is typically used to calculate the surface areas of solids through the physical adsorption of a monolayer of gas molecules (liquid nitrogen or argon) onto the solid surface at a specific temperature and pressure. The BET surface represents the surface area that is freely accessible to gases and it is calculated by determining the number of adsorbed molecules or atoms on the surface and by assuming a cross-sectional area of one adsorbed molecule or atom. This method can provide a particle size but it assumes a monodisperse system of average sized spheres of known density, by neglecting the possibility of having a polydisperse system (it does not account for the size distribution of the particles) (Zhou et al., 2009; Dhawan and Sharma, 2010).

The NP surface composition analysis is generally considered with less importance respect to size, shape, aggregation, agglomeration, etc. However, the role of the surface properties of NPs in their toxicity and how these properties modify during exposure under the influence of different environments need attention, as they govern the way in which particles interact with biological environments (Buzea et al., 2007; EFSA, 2009, 2011). Electron spectroscopies (Auger electron spectroscopy—AES, and X-ray photoelectron spectroscopy—XPS), secondary ion mass spectroscopy—SIM, AFM and scanning tunneling microscopy are some of the analysis techniques that provide information about topography, elemental composition, molecular and chemical state and structure (Baer et al., 2010; Lozano et al., 2013; Brun et al., 2014; Yang et al., 2014).

Particle-Induced X-ray Emission (PIXE), a technique that historically has been used to quantify trace elements in materials, can be used to detect trace metallic contaminants and/or NPs in liquids without sample preparation (Yang et al., 2014). The sample is irradiated with an ion beam (i.e., protons), this causes an emission of X-rays from each present element. The X-rays of each element are emitted in a specific energy range and are proportional to the quantity of such element. PIXE offers several advantages: multi-element acquisition in a single measurement, parts per million (ppm) levels of sensitivity, fast measurements (few minutes) and minimal sample preparation. When compared to similar techniques like energy dispersive X-ray (EDX) spectroscopy, PIXE has a higher sensitivity due to lower background noise (Lozano et al., 2012).

## Applications

The detection, the measurement and the quantification of NPs contained in consumer products such as food, food packaging, cosmetics, and personal care products is particularly challenging. The type of matrix in which the NPs are dispersed (e.g., liquid, partially hydrated or semifluid materials, dry solid, emulsion, syrups, oils, polymer) addresses the experimental design necessary to measure the set of physicochemical parameters required, a choice different case-by-case. A complete NPs characterization might not be feasible in all situations, but the combined use of different methods, such as a size separation technique (e.g., FFF, chromatography) coupled with an identification and quantification technique (e.g., ICP-MS) should give the starting point for deeper analyses.

### Food

**Figure 2:**
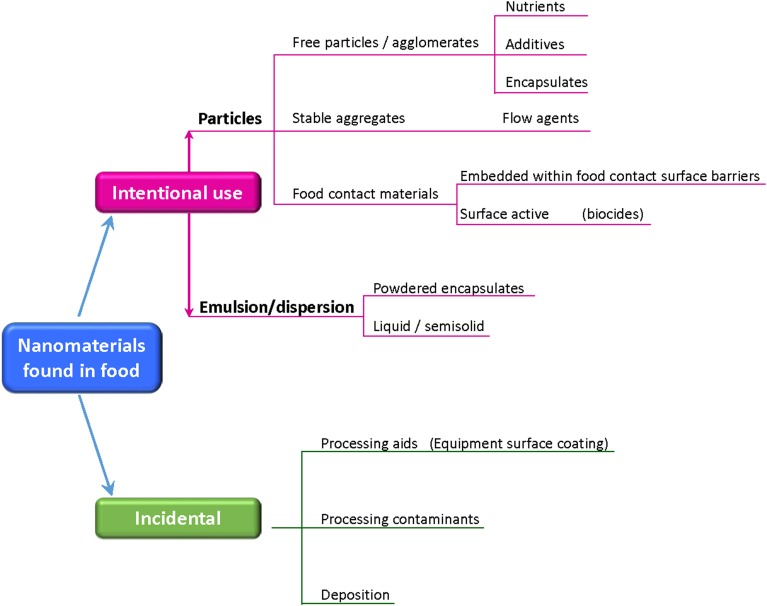
**Nanomaterials which might be found in food**. (Adapted from http://www.riskscience.org).

The research and development of nanotechnologies in the food sector is very active and intense in all steps, from the food processing, to the packaging and delivery. Some food products are now enriched by NPs improving the nutrient and bioactive delivery systems, texture and flavor encapsulation, microbiological control. In the area of food processing and packaging, NPs are employed either as antimicrobial and to build highly sensitive biosensors for detecting pathogens, allergens, contaminants, and degradants that can affect food quality and safety (Magnuson et al., 2011 and reference within). The result of these applications is that many food products, consumed in some cases from centuries and containing naturally occurring NPs, are now enriched by intentionally added or contaminating NPs (Figure 2), and the contamination could have its roots also in the agriculture, where nanoformulations are used to boost the production (pesticides and fertilizers, animal health, animal breeding, poultry production) (Sekhon, 2014).

**Figure 3:**
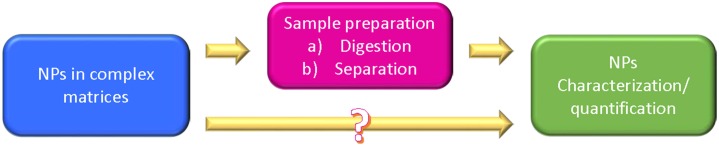
**Possible experimental strategies for analyzing NPs in complex matrices**.

On the consumer safety point to view, the NPs content control should distinguish the native NPs from the intentionally added or contaminating NPs (Hassellov et al., 2008; Blasco and Picò, 2011), with the awareness that ENPs are usually incorporated into foods at low levels. Considering the number of different NPs used in the food and feed sector and their potential interaction with food-matrix components (e.g., proteins), their determination (identification, quantification, and characterization) requires always tailored solutions (OECD, 2012; Blasco and Picó, 2013). Only in very few situations, the food samples could be directly analyzed without some sort of sample preparation, in almost all cases the analytical methods require that NPs be extracted from their native environment, or that the environment be digested, destroyed, or critically altered so that the NPs are in a state that can be measured (Figure 3). This introduces two issues that can compromise the value of the analytical results. First, sample preparation methods are generally not standardized, making difficult to compare results from one laboratory to another with confidence (OECD, 2012). Second, little is known about how the sample preparation impacts on the NP characteristics, so it is difficult to know whether samples that have been prepared following a certain protocol produce data that are a realistic representation of NPs in their native environments (Szakal et al., 2014; Wagner et al., 2015), so that, ideally, methods that avoid or reduce the impact of sampling should be preferred (Tiede et al., 2008; Noonan et al., 2014).

**Figure 4:**
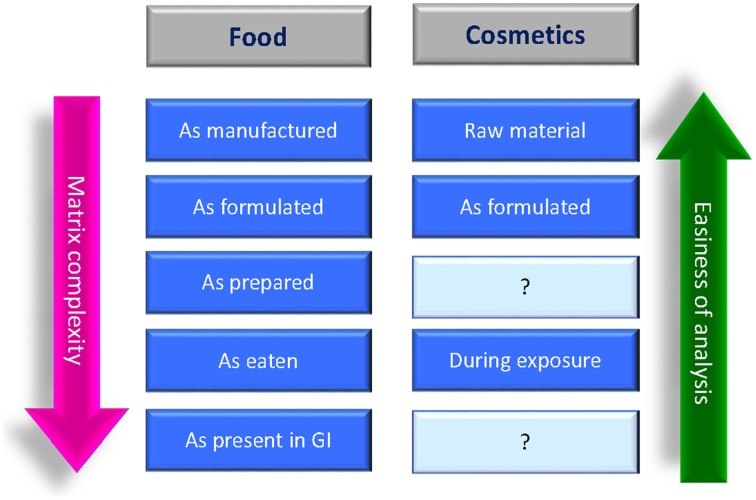
**Suggested stages for the NPs characterization in food/feed products and in cosmetic/personal care products (SCCS, 2012a; Singh et al., 2014)**.

In the field of food products, the characterization of NPs should include five stages: as manufactured, as delivered for use in food/feed products, as present in the food/feed matrix, as used in toxicity testing, and as present in biological fluids and tissues (Figure 4), this because the same physicochemical parameter might change in the different environments. The determination of the physicochemical characteristics of NPs is important in all stages since, for example, as manufactured involve the workers exposure, *in situ* (in the food/feed matrix) is relevant for the toxicity testing and in biological fluids and tissues is important for the “absorption, distribution, metabolism, and excretion” (ADME) studies.

#### What are the NPs of greatest interest in the food sector?

From the list of representative manufactured nanomaterial reported in OECD (2010), the most investigated NPs in the food sector are SiO_2_, TiO_2_, ZnO, and Ag since they are those directly added or indirectly incorporated in food via environmental contamination or migration from food contact materials (OECD, 2009b; Wang et al., 2013).

#### Silicon dioxide SiO_2_

There are various forms of synthetic amorphous silica (SiO_2_) available on the market suitable for food applications. Colloidal silicas are stabilized dispersions of non-agglomerated, mostly spherical SiO_2_ particles used in the food industry as an aid for clarifying wine, beer, fruit juices etc. Precipitated silica is made up of primary particles in the size range of around 5–100 nm, aggregated and agglomerated in the final product; it is used as anti-caking agent in food powders, in health care products such as toothpastes, detergents, and cosmetics. Pyrogenic (fumed) silica consists of agglomerated and aggregated primary particles of size typically between 5 and 100 nm; it is used in cosmetics and toothpastes, as antistatic agent in animal feedstuffs and hygroscopic powders, as carrier for active ingredients, as antifoaming agent in the manufacture of decaffeinated coffee and tea, poultry and seafood processing (Commission staff working paper (EU), 2012).

The amorphous silica used as food additive is classified as E551, and it has been approved in the Annex II of Regulation (EC) (2008), updated by Commission Regulation (EU) (2011), under revision in its nano-form [Commission Delegated Regulation (EU), 2013].

The simultaneous determination of size and concentration of standard SiO_2_-NPs in aqueous suspensions, controlling whole methodology of analysis, with a validate procedure is given in only two papers, the first is based on an on-line AF4-ICPMS method (Barahona et al., 2015), the second is based on the on-line FFF-MALS-ICP-MS/MS method (Aureli et al., 2015). The reason of this scarceness could be the limited availability of certified reference SiO_2_-NPs suspensions of appropriate size and concentration in the nano-range (Institute for Reference Materials and Measurements (IRMM website^3^). Both cited methods were in-house validate, giving recovery, precision, trueness, linearity and limits of detection information and advancing the problem of a possible application to more complex samples. Silica NPs were determined with LOD of 0.16 mg L^−1^ (20 nm) and 0.3 mg L^−1^ (150 nm) in a linear range spanning between 0.1 and 25 mg L^−1^ (Barahona et al., 2015) and with a LOD of 0.2 mg L^−1^ in the range 1–500 mg L^−1^ (Aureli et al., 2015).

SiO_2_ NPs as manufactured, i.e., as ingredient for food application (Figure 4), were sized characterized by Dekkers (Dekkers et al., 2011) and Contado (Contado et al., 2013). Fumed and precipitated SiO_2_ were sized by using a set of techniques (SdFFF, SEM, TEM, and DLS), confirming the presence of primary NPs (7–10 nm) organized in clusters or aggregates of different dimensions, the predominant form in foodstuffs (Contado et al., 2013). The surface area was measure by BET (Dekkers et al., 2011).

Powdered products like milk, instant soups, sauce and seasoning mixes, cake mixes, coffee creamers, and vitamins, are surely the products in which the presence of SiO_2_ as E551 is more probable because of its anticaking properties. When the food product is a relatively simple matrix (e.g., instant coffee), the detection and quantification of SiO_2_ NPs could be done quite easily on the product prepared as the consumer uses (Contado et al., 2013), when the food matrix is more complex, the sampling issue becomes important. Dekker and coworkers (Dekkers et al., 2011) analyzed several food products reporting on their label the E551 as ingredient. The combined use of HDC and ICP-MS allowed to determinate simultaneously the concentration of nanosilica (ranged from < 0.1–1.0 mg g^−1^ product) and the particle sizes, ranging from 50 to 200 nm.

The quantification of the total content of SiO_2_ in a food matrix is usually not a problem, since techniques such as GFAAS, ICP-OES, or ICP-MS are able to get this information after acidic digestion, however this determination does not account of the natural Si contribution neither allows to discriminate between its particulate and ionic forms.

A protocol to extract the silica particles from a simple food matrix was proposed by Contado et al. (2013). A cappuccino powder mix was dissolved first in hot water and then treated with hexane to remove the organic components and the extracted particles were characterized only for their sizes by SEM and SdFFF. A more complete characterization of the E551 NPs isolated from a selected food product was done by Athinarayanan et al. who used XRD, FTIR, TEM, EDX, and DLS to determine the morphology, particle size, crystalline nature and purity (Athinarayanan et al., 2014). Incidentally; this work is interesting also for the toxicological experiments, which have proven that the isolated amorphous nano-sized particles (NPs) (10–50 nm) affect the human lung fibroblast cell viability, intracellular ROS levels, cell cycle phase, and the expression levels of metabolic stress responsive genes. A significant finding for the use of the E551 NPs in the food industry (workers' exposure). Another powerful technique able to quantify SiO_2_ NPs dispersed in water, coffee, and milk is PIXE. This technique offers fast measurements, minimal sample preparation, and ppm levels of sensitivity (Lozano et al., 2012).

A first attempt to detect directly the SiO_2_ NPs in a food preparation was done by Luo et al. who observed by ASEM imaging spherical SiO_2_ NPs in a tomato soup (Luo et al., 2013). The tomato soup was prepared by Dr. T. Linsinger and Dr. R. Grombe of the Joint Research Centre (Brussels, Belgium) as reference material under laboratory conditions for NPs fate studies (Grombe et al., 2014). The particles were arranged in loosely packed agglomerates ranging between 85 and 600 nm (TEM), along with larger agglomerates of few microns (ASEM). The sizes were also determined by NTA, confirming that different analysis methods produce different sizing results.

Fresh fruits and vegetables are other important food matrices. Cultivated surfaces treated with NPs to improve the production or NPs dispersed in the environment may lead to the incorporation of NPs into foods through the food chain. In a recent publication SiO_2_, Al_2_O_3_, and TiO_2_ were used to contaminate the surface of cherry tomato, taken as a food model (Ovissipour et al., 2013). TiO_2_ and SiO_2_ NPs lasted on the tomato surface after a washing treatment with deionized water, while the procedure removed Al_2_O_3_ NPs. The up-take of the NPs on the surface was monitored by ICP-MS and SEM, an the fourier transform infrared (FT-IR) spectrometry determined the spectral changes in the biochemical properties of the tomato surface before and after treatment with a suspension of NPs. The particles were characterized by DLS, either for their sizes and surface charge, this last determined also by electrophoretic light scattering (ELS).

When orally ingested, NPs traverse the digestive tract through the mouth, stomach, and intestines, they will be exposed to a number of digestive enzymes. The safety assessment of SiO_2_ NPs (for example as E551) and the evaluation of their possible toxic effect cannot ignore the possible dissolution and/or agglomeration that particles experiment during the digestion process (Dekkers et al., 2013). Peters and co. considered, as model, three food matrices (hot coffee, instant soup, and pancake); they were added with three forms of silica: E551, SAS (synthetic amorphous silica), and SiO_2_ NPs (32 nm) and the matrices were treated with a sequence of solutions, simulating the human digestion fluids. The results showed that nano-sized silica (5–200 nm) were present in the saliva digestion stage, disappeared during the successive gastric digestion stage in favor of large agglomerates (HDC-ICP-MS, DLS, and SEM data) and it reappeared in the intestinal stage, even in a higher amount respect to the saliva stage because of the neutral pH condition. This indicates that the human intestinal wall is likely the most exposed part of the GI track to the NPs (if the silica concentration in the GI track would be constant and there would not be adsorption on the track) (Peters et al., 2012).

A frequently *in vitro* model for the intestine epithelium is by the Caco-2 cell line, derived from colonic epithelial adenocarcinoma cells. These cells may be used in their undifferentiated and differentiated state. Amorphous SiO_2_ fumed NPs (14 nm, specific surface area of 200 m2g-1) were used to investigate their possible uptake by both types of Caco-2 cells in a recent study (Gerloff et al., 2013). The NPs were treated with solutions of different pH and composition to simulate the gastric and intestinal digestions, avoiding the sonication, to simulate the real digestion process. Size distribution and solubility were not affected by the simulated digestion, but SiO_2_ particles partially lost their ability to generate superoxide anion radicals (${\text{O}}_{2}^{-}$) after the digestion treatment (EPR measurements) and the toxic inflammatory effects in human intestinal Caco-2 cells induced by the SiO_2_ mainly depended on the differentiation status of the cells. The tendency of the SiO_2_ particles to aggregate and agglomerate in the cell culture medium was documented either by TEM and DLS measurements, while the amount of SiO_2_ up-taken by the cells was measured by ICP-OES (Gerloff et al., 2013).

The agglomeration/aggregation status of NPs, as key criterion that may modulate their toxicity, is indicated also by Tarantini et al., underlying as this point requires an in-depth analysis in the framework of toxicity studies (Tarantini et al., 2015). They evaluated the toxicity of 15 and 55 nm amorphous SiO_2_ NPs, compared to microparticles of crystalline silica, on the Caco-2 cell line, proving that the 15 nm NPs were more toxic than the 55 nm NPs or quartz, probably because of the higher surface and the greater number of particles. The primary size and the hydrodynamic diameter of SiO_2_ NPs were characterized by TEM and DLS. Another interesting work, examining in depth this point, was done by Uboldi et al. Some amorphous silica NPs were characterized by TEM, SEM and by DLS (protocols ISO 13321:1996 and ISO 22412:2008). The SiO_2_ NPs were dispersed in deionized water and in the culture media (serum-free medium), observing a little effect on stability of NPs when dispersed in serum-free cell culture medium. SiO_2_ NPs dry powders were difficultly to disperse, and the suspensions maintained an aggregated/agglomerated state, greatly increasing the characteristic size at which they were biologically tested (Uboldi et al., 2012). The aggregation/agglomeration of the SiO_2_ NPs (< 50 nm) in culture media (loading levels of 10 μgcm^−2^) was observed also by McCracken et al.; when treated with simulated intestinal digestive solution, the surface SiO_2_ (and TiO_2_) was covered with bile salts/proteins (IR infrared spectroscopy data), without showing toxicity, but TEM observations documented the SiO_2_ NPs internalization by the C2BBe1 cells, another intestinal epithelial cell line derived as subclone from Caco-2 cells (McCracken et al., 2013).

The findings of the papers just cited stress that the properties of nanosized materials used for safety assessment must be carefully investigated for characterizing their behavior in testing media and to derive reliable interpretation of toxicological data, as recommended also elsewhere (Aureli et al., 2012; OECD, 2012). In addition, they evidence that the surface chemical characterization of SiO_2_ NPs for food applications, is still scarce during the *in vitro* studies, while it should deserve a deeper attention.

#### Silver nanoparticles AgNPs

Silver is a food additive (E174) approved by the European Commission [European Parliament and Council Directive (EC), 1994] and authorized to be used *quantum satis* as a silver-colored powder or as tiny sheets to color the external coating of confectionery, for decoration of chocolates and in liqueurs. Because of its antibacterial action, silver is also allowed in the processing, the conservation, and the consumption of food, e.g., as antibacterial coating of food preparation equipment, storage containers, packaging materials and inner surfaces of fridges and dishwashers, as well as being incorporated into plastic food containers (Verleysen et al., 2015). The presence of E174 as a coloring agent in medicinal products represent an acceptable level of exposure. In 2010, E174 has been included in the program for the re-evaluation of food additives [Commission Regulation (EC), 2010, 2011].

Nano-silver is not therefore considered a food ingredient, even if it might be ingested in the form of dietary supplements, but it might be found in food products as contaminant, as residual of pesticide treatments in agriculture or migrating from containers, knowing that packaging materials containing AgNPs have been commercially available outside the EU since many years (Cushen et al., 2013). Despite silver concentration should be very low, the concern about silver comes from the awareness that AgNPs might have adverse health effects, especially at high doses [Commission staff working paper (EU), 2012]. Silver is usually searched as AgNPs, i.e., in its particulate form since it should stay intact as particle after digestion through the gastrointestinal track (Walczak et al., 2013), however, AgNPs are difficult to be detected since they tend to dissolve in ions and/or aggregate/agglomerate.

Among all the possible analytical methods able to detect and size silver NPs, the most indicated are those showing a high sensitivity and those capable to discriminate the silver species. The reason lies in the low concentration of the silver released from the food containers or the packaging material. The protocol suggested by the EU to measure the released amount of AgNPs, foresees to place in contact the food containers with food stimulant solutions [Union Guidelines on Regulation (EU), 2014] (Table 5). The amount of silver present inside the containers and in the simulant solutions can be determined by ICP-MS (von Goetz et al., 2013; Artiaga et al., 2015), which used in the single particle detection conditions (Laborda et al., 2011), is able to differentiate between the dissolved silver and silver NPs (Echegoyen and Nerín, 2013), or by AAS (Huang et al., 2011). The morphology of AgNPs in the food-simulating solutions can be carried out by TEM (Huang et al., 2011; Song et al., 2011) or by SEM and energy-dispersive X-ray (EDX) analysis of both the test plastics and the simulant extracts (Echegoyen and Nerín, 2013; Artiaga et al., 2015).

**Table 5:** **Food simulants used as from 31-12-2012 (Union Guidelines on Regulation (EU), 2014)**.

**Food simulant**	**Composition**	**Note**
Food Simulant A	Ethanol 10% (v/v)	
Food Simulant B	Acetic acid 3% (w/v)	
Food Simulant C	Ethanol 20% (v/v)	
Food Simulant D1	Ethanol 50% (v/v)	
Food Simulant D2	Vegetable oil	
Food Simulant E	Poly(2,6-diphenyl-p-phenylene oxide), MPPO, particle size 60–80 mesh, pore size 200 nm	Simulant for dry foods

For a more reliable toxicological assessment, the AgNPs migration should be measured into a real food system. Skinless, boneless chicken breast meat is proposed as food matrix because is a valuable, perishable food, and it should benefit of antimicrobial packaging (Cushen et al., 2013). This more realistic experiment complicates the analysis procedures. The total amount of silver can be still measured by ICP-MS but after an acidic digestion of the food matrix (Figure 3); this implies that silver quantification cannot distinguish the particulate and the ionic form (Cushen et al., 2013, 2014). If a reasonable amount of AgNPs is extracted from the food matrices, the evaluation of the Ag^+^/Ag^0^ ratio (molar ion to silver NPs) could be done by using the hollow-fiber flow FFF (HF5) and multi-angle light scattering (MALS), an experimental approach which allows also to determine the dimension and the shape of the dispersed AgNPs in aqueous media (Marassi et al., 2015).

FFF, HDC, and DLS are all good methods to determine the size distributions, but they require to have the NPs suspended in solution prior to testing. Loeschner et al., following the suggestions of Linsinger (Linsinger et al., 2013), set up a method based on the use of the AF4 coupled with the ICP-MS, used in single particle mode. The chicken meat matrix was spiked with a known amount of roughly 40 nm AgNPs, enzymatically digested (Proteinase K) and the released AgNPs were detected and sized into a liquid suspension (Loeschner et al., 2015). TEM was also used to get complementary size information (Loeschner et al., 2013; Szakal et al., 2014). Unfortunately, this procedure cannot be applied to long-term frozen chicken meat, since the AgNPs are instable in terms of dissolution, chemical transformation and agglomeration/aggregation.

AgNPs were also detected and sized in four nutraceuticals and a beer products by AF4-ICP-MS (LOD of 21 and 28 ngL^−1^). The sample preparation was indicated as the most delicate step during NP analysis because of the difficulty to keep the NPs in solution as separate entities, due to the coexistence of other matrix components, which promote NPs aggregation or adsorption. The total amount of silver was determined by ICP-MS after microwave-assisted acid digestion, while the dimensions were confirmed by TEM (Ramos et al., 2014).

To reduce the sample preparation time down to 1–2 h and avoiding particular skills for thin sectioning of frozen or embedded material, Lari et al. proposed a simplified sample preparation protocol to determine AgNPs in meat samples based on TEM (Lari and Dudkiewicz, 2014). The AgNPs/meat viscous liquid achieved by spiking the meat emulsion (Grombe et al., 2015) with 42 nm AgNPs, was diluted, homogenized and sediment by ultracentrifugation on TEM grids. The meat sample thickness was determined by energy filtered transmission electron microscopy (EFTEM) and electron energy loss spectrometry (EELS). Advanced TEM methods and conventional and single particle—ICP-MS were used also to detect AgNPs released by colored pearls meant used for the pastry decoration (Verleysen et al., 2015).

A different approach to detect the AgNPs directly in the food matrix could take advantage of their characteristic surface plasmon resonance. Rebe Raz et al. (2012) showed as a favorable protein ligand can capture AgNPs creating a hMT1A-sensor with a sensitivity in the microgram-per-liter range, displaying the highest sensitivity toward larger and uncoated AgNPs. This method requires a selective sample-preparation for nanoform confirmation because of the possible cross-reactivity toward ions. This sensor detected AgNPs in fresh vegetables and river water extracts, within 10 min, without the need in complex sample preparation steps.

Fresh fruits and/or vegetables represent another type of food matrix where the AgNPs might be found as residual of agricultural treatments (e.g., pesticides based on silver NPs). To evaluate the possible surface penetration of AgNPs (20 and 70 nm) into pears tissues Zhong and co. used the TEM, SEM and ICP-OES techniques; DLS and EDS determined instead size, shape and other properties of AgNPs in solution or in pear tissues, demonstrating that the 20 nm AgNPs penetrated the pear skin and pulp (Zhong et al., 2012).

#### Titanium dioxide TiO_2_

Food grade TiO_2_ is coded in EU as E171 and its specification for food uses is in Commission Directive (2009), which update the Commission Directive 95/45/EC. In Europe its use in foods is permitted in general, with some specified exceptions, at *quantum satis* levels (i.e., as much of the substance that is needed for the desired effect, but not more), while in the USA, the use of TiO_2_ as a human food additive must not to exceed 1% by weight (FDA CFR website^4^).

TiO_2_ is used as a color additive (brilliant white, color index CI 77891, Pigment White 6) in human food products because of its brightness, high refractive index (>2.4) and as a texture modifier in a wide variety of confectionary foods, toothpastes, and other ingestible products (cottage and mozzarella cheeses, horseradish cream and sauces, lemon curd, and in low-fat products such as skimmed milk and ice-cream). TiO_2_ is frequently declared as a “natural coloring agent” and is therefore well accepted by consumers. TiO_2_ is also used in oral pharmaceutical formulations, and the Pharmaceutical Excipients handbook (Rowe et al., 2003) considers nano-sized TiO_2_ a non-irritant and non-toxic excipient.

Information about the physicochemical properties of nanosized TiO_2_ for food applications is limited even though the number of products containing it is considerable and increasing; no information is usually given about the quantity, particle size and particle structure even when the product is labeled as containing E171.

Yang and coworkers have analyzed P25 and five TiO_2_ powders, available on the market as food additives (raw materials), to provide information about their chemical composition (ICP-MS), surface functionality (XPS), morphology (TEM), particle size distributions (DLS), crystal structure (XRD and Raman spectroscopy), size and surface charge in water (ZetaPALS), and photoactivity (diffuse reflectance; solar activity toward organic dyes). All five TiO_2_ food-grade samples contain particles smaller than 100 nm, an evidence that could pose workplace exposure hazards (Yang et al., 2014).

The TiO_2_ concentration in a wide range of white food products (89), some labeled as containing TiO_2_ (E171) other not, was determined by ICP-MS after acidic digestion of the samples, and the particle sizes were measured by TEM and DLS (Weir et al., 2012). The foods with the highest content of TiO_2_ were candies, sweets and chewing gums, as confirmed also by Chen et al., who focused his investigations on sugar-coated chewing gum, with a well detailed qualitative and quantitative study, which assessed that over 93% of TiO_2_ in gum is nano-TiO_2_, and it is unexpectedly easy to come out and be swallowed by a person who chews gum. The extracted NPs were characterized for their chemical composition, morphology, size distribution, crystalline phase, particle and mass concentration, surface charge, and aggregation state (Chen et al., 2013).

Dietary supplements is another emerging market where micrometer-sized TiO_2_ and SiO_2_ are commonly utilized. Lim et al. has examined 12 dietary supplements (marketed specifically for women) claiming the inclusion of TiO_2_ and SiO_2_, without providing any information about the particle size. SiO_2_ and TiO_2_ particles were selectively separated from the products using a simple high-temperature acid digestion and centrifugation step, in which other metallic particles were dissolved during the process. The particles, characterized by DLS, FESEM, TEM EDX XRD for their average size, morphology, size distribution, and crystal structure, were found usually aggregated and 11 products over 12 contained nanometer-sized particles (Lim et al., 2015).

AF4-ICP-MS, SEM, and single-particle ICP-MS (sp-ICP-MS) techniques were used to compare the number-based particle size distributions of several types of TiO_2_ food additive E171, food products such as cakes, candy, and chewing gum and personal care products such as toothpaste. All methods provided comparable (number-based) size distributions, indicating that these methods can reliably be used to enforce food labeling in line with the recommendation of the EU definition of nanomaterials (Peters et al., 2014).

As already mentioned, fresh fruits and vegetables, if during their cultivation and ripening become in contact with NPs, they are able to translocate the NPs in their tissues and accumulate them (Servin et al., 2012). If the translocation process interests also the edible parts, NPs enter in the food chain. Cucumbers harvested on a soil treated with semispherical TiO_2_ NPs (27 ± 4 nm) were analyzed by synchrotron μ-XRF and FTIR techniques, proving that cucumber plants take up both anatase and rutile crystalline phases from the soil and transport them through the tissues without crystal phase modification. The ICP-OES analysis showed that cucumber fruit from plants treated with 500 mg TiO_2_ kg^−1^ NPs are able to modify the content of the primary macronutrients (Servin et al., 2013).

The determination of TiO_2_ NPs in organ tissues is rather challenging since the metal analysis would require all the metal be fully dissolved (digestion) while the detection of NPs should not destroy them. A good example of inter-laboratory comparison, based on ICP-MS, is reported by Krystek et al., where the digestion was done selecting appropriate acids and heating systems (Krystek et al., 2014). An alternative protocol for measuring Ti from TiO_2_ NPs in the tissues of fish, performing an incomplete digestion and using unmodified ICP-OES instrument to simultaneously measure other elements has been proposed by Shaw et al. The method improved the recovery of Ti metal from TiO_2_ NPs and the reproducibility of the analysis (Shaw et al., 2013).

The aggregation state of TiO_2_ NPs (nominally < 80 nm) dispersed in BSA was successfully observed by ASEM by Luo et al. (2013).

The potential hazard of exposure to nano-sized TiO_2_ for humans and environment has been recently reviewed by Skocaj et al. in regard to the particle size and the crystal structure of TiO_2_ (Skocaj et al., 2011). A possible consequence of TiO_2_ NPs exposure for ingestion is the disruption of the epithelial surface brush border of the absorptive cells of the intestine. If this happens, the surface area assigned to the food adsorption decreases with malnutrition consequences. A biological effect caused by food grade TiO_2_ NPs was observed also in another *in vitro* test on Caco-2BBe1 cells. TiO_2_ NPs isolated from the candy coating of gum and well characterized by the XPS, XRD, TEM, and DLS techniques, apparently elicit a *bona fide* biological response and not simply a physical artifact as a consequence of *in vitro* exposure (particle sedimentation) (Faust et al., 2014).

#### Zinc oxide ZnO

ZnO is listed as “generally recognized as safe” (GRAS) by the U.S. FDA (21CFR182.8991). As a food additive, it is the most commonly used zinc source in the fortification of cereal-based foods. ZnO has been also incorporated into the linings of food cans in packages for meat, fish, corn, and peas to preserve colors and to prevent spoilage. The current search work for finding effective biocidal agents, alternative to the high costly gold and silver, is focusing on metal oxides and ZnO, in its nanoparticulate form, is a good candidate for the development of novel food packaging products (Chaudhry et al., 2008; Tankhiwale and Bajpai, 2012) because of its antimicrobial and UV-absorbent properties (Xie et al., 2011).

The physicochemical characterization of ZnO NPs for food applications is still very scarce, only few studies have focused on the migration of ZnO nanoparticles to food and the toxicological impact of ZnO NPs must still be evaluated to determine the positive or negative effects on food safety (Espitia et al., 2012).

### Cosmetics and personal care products

The cosmetics sector has more products than the food sector claiming or even positively advertising the use of nanomaterials, even if sometimes it is unclear whether nanomaterial are really used or if companies just use the term “nano” for advertising purposes. Nevertheless, the number of research papers regarding the characterization of NPs used in the cosmetic products is inferior respect that about food products, so much so that a recent review has hoped that the use of nanotechnology in cosmetic products be more transparent to facilitate realistic risk assessments (Wiechers and Musee, 2010).

The use of nanomaterials in cosmetic products in Europe is regulated by the EU Cosmetics Regulation No 1223/2009 [Regulation (EC), 2009], which provides a definition of nanomaterial, as well as a mechanism for notification, labeling, and safety evaluation of cosmetic products containing nanomaterials. The Regulation covers mainly those nanomaterials that are intentionally made and are insoluble/partially-soluble or biopersistent (e.g., metals, metal oxides, carbon materials, etc). For these nanomaterials the characterization must include the measurement of the physico-chemical parameters listed in the SCCS Guidance on the Safety Assessment of Nanomaterials in Cosmetics (SCCS, 2012a), the same suggested also by the European Food Safety Authority (EFSA, 2011) and summarized in Table 2. The characterization must be carried out on the nanomaterial at the raw material stage, in the cosmetic formulation, and during exposure for toxicological evaluations, but to facilitate the risk assessment further information regarding the description of production processes, any surface modifications, and the preparatory steps carried out for integrating the nanomaterials in the final cosmetic products could be necessary (SCCS, 2012b) (Figure 4).

NPs in cosmetic products cover the product formulation and the packaging. In the formulation sector, NPs are used as active substances, carriers and formulation aids with the aim to enhance the efficacy of the product (BEUC, 2012). Sunscreens with efficient UV protection, long-lasting make-up, anti-aging creams with an increased intake of vitamins or enzymes, toothpaste and hair care or coloring products are only few examples of products, which might contain NPs.

#### Titanium dioxide TiO_2_ and zinc oxide ZnO

The highest content of TiO_2_ in cosmetic and personal care products might be found in toothpastes and sunscreens (1 to >10% titanium by weight), while shampoos, deodorants, and shaving creams contain the lowest levels of titanium (< 0.01 μg mg^−1^). For several high consumption pharmaceuticals, the titanium content ranged from below the instrument detection limit (0.0001 μg Ti mg^−1^) to a high of 0.014 μg Ti mg^−1^.

Zinc oxide is also used as bulking, skin protector and as an UV absorber; in Europe it is authorized also as colorant (color index CI 77947) in Council Directive 76/768/EEC Annex IV) in all cosmetics.

Sunscreens are likely the best known products containing TiO_2_ and ZnO NPs because of their action as ultraviolet (UV) filters (Serpone et al., 2007). The most modern sunscreen formulations contain now colorless TiO_2_ and ZnO particles, i.e., nano-sized which filter UV light more efficiently than their corresponding micron-sized particles. The typical size of TiO_2_ NPs in sunscreen ranges now from 10 to 100 nm (Nohynek et al., 2007). TiO_2_ and ZnO NPs formulated in topically applied sunscreen products exist as aggregates of primary particles ranging from 30 to 150 nm in size. These aggregates should not modify their structure once applied on the skin, nor release primary particles (Schilling et al., 2010), so that, based on the current, limited available data, the risk for humans from the use of nano-structured TiO_2_ and ZnO in cosmetic preparations or sunscreens should be considered, by some researchers, negligible. Unfortunately, it was demonstrated also that micronized ZnO is photoclastogenic, possibly photo-aneugenic, and a photo-DNA damaging agent in mammalian cells cultured *in vitro* (McCall, 2011; SCCS, 2012c). Osmond et al. (Osmond and McCall, 2010) discussed the potential human exposure and the health hazard at each stage of manufacture and use of the ZnO NPs destined for use in modern sunscreens, concluding that there is a need for further research, deeper studies and appropriate investigations *in vivo*.

This advice should be considered especially knowing that TiO_2_ and ZnO NPs, specifically for cosmetics, are usually coated with substances to enhance their compatibility with lipophilic components of cosmetic applications. These coatings alter the particle surface hydrophilicity/hydrophobicity and with it the biological reactivity, the penetration depth and the intracellular particle distribution (Teubl et al., 2015). For these and other inherent problems, the safety assessment of TiO_2_ and ZnO NPs calls for a much deeper investigation (Newman et al., 2009; Nohynek et al., 2007).

Studies about the physicochemical characterization of TiO_2_ for cosmetic are not numerous, but more than for ZnO, which are completely absent. The quantification of TiO_2_ in sunscreens is done traditionally with spectroscopic techniques such as GFAAS, FAAS, or ICP-AES (Mason, 1980; Salvador et al., 2000; Zachariadis and Sahanidou, 2009), even if a recent publication reports the TiO_2_ determination in 15 commercial samples achieved with a portable energy dispersive X-ray fluorescence (EDXRF) without sample preparation (Melquiades et al., 2008).

The techniques suitable to give size information of the NPs are basically the same used in the food sector. A short list of them is reported by Mu et al. (Mu and Sprando, 2010), even if recent publications have indicated the FFF techniques particularly useful to achieve these data (Contado and Pagnoni, 2008; Samontha et al., 2011), especially when coupled on or off-line with spectroscopic techniques. TiO_2_ NPs in cosmetic and food products were quantified by AsFlFFF–ICP-MS without the need of additional strategies and avoiding the use of calibration standards of different nature (López-Heras et al., 2014). However, this highly promising approach has some instrumental limitations, such as the need to re-equilibrate the membrane in order to avoid the accumulation of NPs, or the limited mass sensitivity due to the high sample dilution occurring during migration in the channel up to detector.

## Conclusions

The characterization of NPs in complex matrixes, such as food and in cosmetic/personal care products, needs much more work to standardize and validate the analytical methods required to determine those physicochemical parameters important to describe the biological interactions.

From this overview of recently published papers emerges the general need of creating reference materials and developing reference protocols. A satisfying characterization of the NPs used in these two important industrial sectors is available only for the lowest step (as manufactured) of the five suggested stages (Figure 4), mainly because of the increasing complexity of the matrices, passing from the pristine NPs to the commercial end products or to the biological substrates.

The surface chemical characterization of the NPs is still scarce in most of the *in vitro* studies, while it should deserve a deeper attention, being the cause of their behavior in testing media (aggregation/agglomeration) and of their biological activity (toxicity).

In this contest, cosmetic products are the less studied and among the metal and metal-oxide NPs here considered, ZnO is the less characterized, either in the food and in the cosmetic field.

Finally, on the regulatory point of view, for a more safety exchanges of products across all Countries, it should be desirable to define a worldwide standardization of terminology.

### Conflict of interest statement

The Guest Associate Claudia Cascio declares that, despite having collaborated with author Catia Contado, the review process was handled objectively and no conflict of interest exists.

## Footnotes

^1^FDA website (U.S. Food and Drug Administration): FDA's Approach to Regulation of Nanotechnology Products http://www.fda.gov/ScienceResearch/SpecialTopics/Nanotechnology/ucm301114.htm Last accessed May 2015.

^2^ISO (International Organization for Standardization) website: ISO/TC 24/SC 4 “Particle characterization” Standards catalogue http://www.iso.org/iso/standards_development/technical_committees/other_bodies/iso_technical_committee.htm?commid=47176 Last accessed May 2015

^3^IRMM (Institute for Reference Materials and Measurements) website. Reference Materials (RM), Geel, Belgium - https://ec.europa.eu/jrc/en/reference-materials

^4^FDA CFR (U.S. Food and Drug Administration - Code of Federal Regulations Title 21) website http://www.accessdata.fda.gov/scripts/cdrh/cfdocs/cfCFR/CFRSearch.cfm?CFRPart=73 (Last accessed May 2015).
